# Perinatal events and development of juvenile idiopathic arthritis-associated uveitis

**DOI:** 10.1038/s41598-023-44208-1

**Published:** 2023-10-16

**Authors:** Aysha Chaudhary, Manahil Nadeem, Jack Townsend, Victoria J. Miller, Amir R. Hajrasouliha

**Affiliations:** Eugene and Marilyn Glick Eye Institute and Department of Ophthalmology, Indiana University School of Medicine, 1160 W Michigan St, Indianapolis, IN 46202 USA

**Keywords:** Autoimmunity, Paediatric rheumatic diseases

## Abstract

Uveitis is one of the most common manifestations of juvenile idiopathic arthritis (JIA). Currently, JIA is associated with decreased gut microbiota diversity. Studies confirm that perinatal events can cause aberrant microbial colonization. The objective of this study is to determine if JIA is associated with perinatal events with a secondary focus on these variables to the development of JIA-uveitis. 369 patients with strabismus (n = 200) or JIA (n = 196) were included in the study. Completed surveys (JIA 37; strabismus 18) collected data about birth route, pregnancy and labor complications, JIA medications, and the presence of eye disorders. Analysis indicates that there is no relationship between JIA development and the perinatal events investigated. Similarly, no significance was found between JIA-uveitis and birth route or labor complications. Pregnancy complications, namely gestational diabetes (GD), were statistically higher in the JIA group with uveitis compared to JIA without uveitis. The data from this survey study showed that JIA-uveitis was highly associated with pregnancy complications, particularly with GD. However, no statistically significant association was found between JIA and route of delivery, labor complications, or pregnancy complications. Further studies are needed to understand the ways that GD interrelates with the development of uveitis in JIA patients.

## Introduction

Juvenile idiopathic arthritis (JIA) is the most common arthritic affliction within the pediatric population^1, 2^. The autoimmune disorder can manifest with symptoms of joint pain, stiffness, inflammation within joints, and an overall sense of malaise. In addition to its rheumatologic symptoms, JIA exhibits ocularimplications where uveitis—an inflammation of the middle layer of the eye—serves as one of the most common extraarticular manifestations^1–3^ with arthritic symptoms typically preceding the development of uveitis^1^.

Patients with JIA associated uveitis (JIA-uveitis) often require long-term immunosuppression and close follow-ups^4^ with the co-occurrence of these disorders predicting a poor overall prognosis^2^. Although well documented as a common co-occurrence with JIA, the pathogenesis, etiology, and predisposing factors of JIA-uveitis remain unclear^4, 5^. As such, the central objective of this investigation is twofold: first, to uncover factors, specifically perinatal, that are linked to the development of both JIA and JIA-uveitis, and second, to lay the foundation for subsequent investigations into the underlying cause of this condition.

Much like several other autoimmune disorders^6–12^, JIA demonstrates a correlation with microbiota dysbiosis which plays a vital function in conferring host immunity^13^. Particularly noteworthy is the reduced concentration of four types of short-chain-fatty-acid-producing bacteria^14, 15^ within the gut microbiota of JIA patients. This reduction in bacterial diversity potentially triggers an altered immune response to the microbiota, contributing to the augmented circulation of auto-antibodies^16^. Although not innately pathogenic, these findings of auto-antibody production offer insight into the potential mechanisms that underlie the developmentof JIA.

Perinatal events, including cesarean section (CS) deliveries and complications during pregnancy or labor are known to modify the microbiotic diversity of an infant. In contrast to vaginal delivery, CS delivery does not facilitate the transmission of maternal microbiota to the child, preventing bacterial colonization and growth^4^. Despite existing research, there remains to be a significant research gap pertaining to CS delivery, perinatal complications, and the subsequent development of JIA or JIA-uveitis.

Thus, the objective of this study was to determine the presence of an association between these variables. In addition to investigating the risk of JIA development with CS delivery, this study also encompasses the examination of other perinatal factors, including complications during pregnancy and labor (Supplementary table).

## Results

### Respondent demographics

Out of the 396 patients meeting inclusion criteria, 200 were included in the strabismus group and 196 in the JIA group. Responses were received from 56 patients via mail—18 from the strabismus group and 38 from the JIA group. Respondent data were categorized based on initial grouping: JIA (n = 38; response rate 19%) and strabismus (n = 18; response rate 9%). Demographics and pathologies collected from each cohort are listed in Tables 1 and 2, respectively. One JIA survey was excluded due to incomplete responses on key variables. The strabisumus group had an average gestational age of birth was 38 weeks, while the JIA group had 39 weeks. Respondents who indicated “full term” (n = 4 JIA; n = 0 strabismus) were assumed to have reached 40 weeks of gestation. JIA patients age of diagnosis ranged from 1 to 15 years, with a median of 6 years.

**Table 1:** Gender, race, and birth route classification of JIA and strabismus survey respondents.

	JIA † number (%)	Strabismus ‡ number (%)	*P* value
Gender			
Males	9 (24%)	11 (61%)	0.018*
Females	28 (76%)	7 (39%)	
Birth route			0.868
Vaginal birth	26 (73%)	13 (72%)	
C-section birth	11 (30%)	5 (28%)	
Race			
White	33 (89%)	16 (89%)	0.183
Asian	0 (0%)	1 (6%)	0.327
Black	1 (3%)	1 (6%)	1.000
Hispanic	0 (0%)	0 (0%)	1.000
Other	3 (8%)	0 (0%)	0.543
Total	37 (100%)	18 (100%)	

Values are expressed as numbers and percentages as compared to the respective cohort population.

Significant values are in italics.

*P* values for the association between the observed variables were determined with Chi-square or Fisher exact testing.

n^†^ = 37.

n^‡^ = 18.

**Table 2:** Pregnancy and Labor Complications in JIA and Strabismus Survey Respondents.

	JIA^†^ number (%)	Strabismus^‡^ number (%)	*P* value
Pregnancy complications	12 (32%)	9 (50%)	0.336
HTN	2 (5%)	4 (22%)	0.157
Gestational Diabetes	7 (19%)	2 (11%)	0.729
Preeclampsia	0 (0%)	2 (11%)	0.103
Anemia	0 (0%)	2 (11%)	0.103
Infection	0 (0%)	1 (6%)	0.327
Breech position	4 (11%)	2 (11%)	0.183
Cigarette smoke	0 (0%)	3 (17%)	0.03*
Alcohol use	0 (0%)	0 (0%)	1.000
Preterm labor	4 (11%)	3 (17%)	0.33
Labor complications	14 (38%)	9 (50%)	0.571
Breech position	4 (11%)	1 (6%)	0.892
Preterm rupture	1 (3%)	1 (6%)	0.812
Preterm labor	4 (11%)	3 (17%)	0.857
De-accelerating fetal heart rate	1 (3%)	4 (22%)	0.188
Placental abruption	1 (3%)	0 (0%)	1.000
Prolonged labor	2 (5%)	3 (17%)	0.388
Umbilical cord prolapses	1 (3%)	0 (0%)	1.000
Post-term pregnancy	1 (3%)	0 (0%)	1.000
Other	2 (5%)	4 (22%)	

Significane of male to female difference between the group, JIA is known to be more common in female than male; hence this significant difference is anticipited between the groups.

Values are expressed as numbers and percentages as compared to the respective cohort population.

*P values for the association between the observed variables were determined with Chi-square or Fisher exact testing.

n^†^ = 37.

n^‡^ = 18.

### JIA development compared with strabismus

Chi-square analysis with Yates correction and Fisher exact test for smaller sample sizes were used to calculate *p*-values for JIA development relative to birth route, pregnancy complications, and labor complications, using the strabismus group as reference. However, no statistical relationship were observed across the variables measured (Table 3).

**Table 3:** Chi-square analysis of JIA and strabismus development with different birth routes and pregnancy/labor complications.

	JIA^†^	Strabismus ‡	*P* value
*Birth route*			
C-Section	11 (0.30)	5 (0.28)	0.868
Vaginal delivery	26 (0.70)	13 (0.72)	
*Complication*			
Labor + Pregnancy	8 (0.26)	4 (0.22)	0.708
Pregnancy	12 (0.32)	11 (0.61)	0.083
Labor	14 (0.38)	9 (0.50)	0.766

Values are expressed as numbers and percentages as compared to the respective cohort population.

*P values for the association between the observed variables were determined with Chi-square or Fisher exact testing.

n^†^ = 37.

n^‡^ = 18.

### JIA-uveitis and complications

Chi-square analysis with Yates correction and Fisher exact test for smaller sample sizes were used to calculate *p*-values for uveitis development in JIA patients with relation to birth route, pregnancy complications, and labor complications using non-uveitis patients as a comparative. Only pregnancy complications indicated a statistical significance to JIA-uveitis development (Table 4).

**Table 4:** Chi-square analysis of JIA with uvetitis development with different birth routes and pregnancy/labor complications.

	JIA with Uveitis^†^	JIA no Uveitis^‡^	*P* value
*Birth route*			
C-Section	0 (0.00)	11 (0.35)	0.151
Vaginal delivery	6 (1.00)	20 (0.65)	
*Complication*			
Labor + Pregnancy	3 (0.50)	6 (0.19)	0.109
Pregnancy	5 (0.83)	7 (0.23)	0.014*
Labor	3 (0.50)	11 (0.35)	0.832

Values are expressed as numbers and percentages as compared to the respective cohort population.

*P values for the association between the observed variables were determined with Chi-square or Fisher exact testing.

n^†^ = 6.

n^‡^ = 31.

### JIA-uveitis and gestational diabetes

Among JIA-uveitis patients with pregnancy (n = 6), gestational diabetes affected 50% of patients (n = 3). This was statistically significant compared to JIA patients without uveitis and experiencing gestational diabetes, as shown by chi-square analysis (Table 5; *p* = 0.033).

**Table 5:** Chi-square analysis of JIA with uveitis development with gestational diabetes.

	JIA-Uveitis †	JIA only ‡	*P* value
*Complication*			
Gestational diabetes	3 (0.50)	4(0.13)	0.033*
No gestational diabetes	3 (0.50)	27 (0.87)	

Values are expressed as numbers and percentages as compared to respective cohort population.

*P values for the association between the observed variables were determined with Chi-square or Fisher exact testing.

n^†^ = 6.

n^‡^ = 31.

## Discussion

Our study stands as a pioneering effort to systematically evaluate variables of pregnancy and labor to determine their association with the development of JIA and JIA-uveitis.

The microbiota hypothesis postulates that CS delivery would decrease overall immune functionality through a combination of aberrant cytokine release and leukocyte recruitment^13, 14^. Accordingly, our study’s underlying assumption was that patients with JIA, which is characterized by decreased variety of microbiota^9, 13–16^, might display an increased liklihood of undergoing CS delivery. However, our results refuted this hypothesis, instead suggesting more studies are needed to unravel the potential impacts of CS delivery on JIA.

The current literature landscape investigates the relationship between CS delivery and autoimmune disorders. With current studies citing associations between CS delivery and inflammatory disorders, asthma, eczema, and connective tissue disorders^17–20^. To note, Yerlikaya et al.^20^ highlighted a significant alteration in the expression of various miRNA sequences in the microbiota of CS patients experiencing inflammatory disorders.

However, the existing body of research also presents evidence that challenges this association. Soullane et al.^21^ is a longitudinal study of 934,873 children that found no statistically significant relationship between CS delivery and the development of autoimmune disorders. The findings of, Räisänen et al.^22^ also corresponded to those of Soullane et al.^21^ with no evidence of CS delivery influence on autoimmune disorders. The paradoxical outcomes underscore the potential complexity underpinning the relationship amongst the factors.

Among such factors, Räisänen et al.^22^ observed that a high proportion of children born of CS delivery were preterm and received both a greater number and frequency of postnatal antibiotics compared to preterm children of vaginal delivery. Antibiotic use can disrupt the intestinal microbiome by depleting the bacterial populations, thus reducing the microbiome’s density^16, 23^. Several studies have documented the emergence of various disorders, including asthma, allergies, inflammatory bowel disorders, eczema and weight gain, to antibiotic use in and its subsequent disruption on the microbiome homeostasis^23–25^. The role of early exposure to antibiotics, therefore, may provide an intermediate factor that accounts for the findings in our study.

To the same effect, differences in breastfeeding amongst CS delivery patients may also provide an explanation for the observed findings^23^. The impact of diet on an infant's microbiota diversity and density can play an intermediate role in autoimmunity. Ongoing studies have postulated that mothers undergoing CS delivery experience a delay in the onset and reduced volumes of breast milk during lactation^23^. This delay curtails the intake of beneficial microbes, namely *Bifidobacterium* and *Lactobacillus* typically found in abundance in breast milk^26–29^. Consequently, this delay may hinder the stimulation of intestinal flora implicated in immunity and autoimmune response.

While our study did not encompass postnatal antibiotic use or breastfeeding habits in its data collection, this opens an avenue for future research and endeavors. Overall, the findings of this study alone cannot discredit the effect of CS delivery on autoimmunity, as observed in numerous previous studies. Rather than discrediting the association between CS delivery and JIA development, the absence of a direct link could be attributed to intermediary factors, such as antibiotic use and breast feeding, affecting bacterial colonization. Further analysis of these factors coupled with a more mechanistic approach to examining microbiota in the disorder development are needed to provide comprehensive reasoning for the observed relationships.

Analysis of complications during labor and pregnancy failed to illustrate any statistical significance (Table 3). This surprising finding contrasted preexisting data supplied by Shenoi et al.^30^, Chaudhari et al.^31^, and Carlens et al.^5^, which found increased risk of autoimmune diseases, such as JIA with various complications. Both Shenoi et al.^30^ and Chaudhari et al.^31^ reported that pre-term delivery was associated with increased risk for JIA development. On the other hand, Carlens et al.^5^ found a borderline statistically significant increase in risk for JIA development in premature children and patients delivered by c-section. Although the differences in results may be a result of our study’s limitations, these findings may be explained by more recent work conducted by Räisänen et al.^22^ and Soullane et al.^21^ which make the case that early postnatal antibiotic and breastfeeding delays, respectively, use potentially influences autoimmune disorder development in preterm children. None of the aforementioned studies included antibiotic use in their analysis, thus a definitive statement of antibiotic use and the relationship of complications during pregnancy or labor is difficult to conclude.

Environmental factors such as maternal alcohol and nicotine use as well as TORCH infections have been known to disrupt fetal development resulting in various birth defects and lowered health outcomes^5, 32^. Our study cohort encompassed a limited number of individuals exposed to maternal alcohol and infection. As such, no statistically significant relationship was calculated. Maternal smoking, however, was a statistically significant factor between the JIA and strabismus groups (Table 3), implying a potential role in their development. This finding aligns with the conclusions made by Colebatch et al.^33^ who suggested the positive relationship between environmental factors, including maternal smoking, on the development of rheumatoid arthritis (RA). The link between RA and maternal smoking found in this study may suggest a link between other forms of autoimmune diseases, such as JIA, and maternal smoking as well.

Although our study did not establish a direct relationship between pregnacy complications and JIA development, there was a positive association between gestational diabetes (GD) and uveitis within JIA patients, potentially suggesting a correlation between these factors. Surprisingly, the effects of GD were confined to its influence on uveitis in JIA patients, with no overall relationship to JIA development.

Children born with GD have an increased risk of adverse perinatal outcomes including hypoglycemia, macrosomia, and birth trauma^34^. The adverse outcomes associated with GD can increase lifetime risk of obesity as well as cardiometabolic diseases^34, 35^. Changes in adiposity observed with children born of GD contribute to the increased risk of lifetime obesity in these patients^36–39^. Babu et al.^35^ found that obese mothers are more prone to present with GD during pregnancy which, through a process of hyperglycemia-induced fetal programming, is associated with increased neonatal adiposity^36, 37^. Muhammed et al.’s^39^ experimental studies indicated that increased fat composition exacerbated autoimmune uveitis in mice models. This was due to the release of proinflammatory mediators with targeted ocular antigens coupled with decreased regulatory immunity that is associated with white adipose tissue^27, 28^. However, a more direct analysis of the association between GD and uveitis would be needed to understand the mechanism for this relationship.

A significant constraint of this study lay in the diminished response rate which, consequently, led to a small sample size and reduced statistical power. Although most variables analyzed indicated a broad range, the study lacked racial diversity with very few non-white respondents. This is an inherent disadvantage of survey studies with little means of improvement. The study’s sample group exhibited a substantial homogeneity in terms of its size and composition, highlighting the necessity to validate the findings through subsequent research before extending them to a broader population. Nevertheless, the study provides the framework and preliminary results that can guide future studies.

## Methods

### Study design

This was a survey-based study including patients diagnosed with JIA, while patients diagnosed with strabismus constituted the control group, selected for comparable age and lack of immunologic association. Inclusion criteria were pediatric and adult patients with a diagnosis of either strabismus (control) or JIA at the Indiana University Health System (Indianapolis, Indiana, USA). Eligible patients were sent surveys via mail, with instructions to return them using the provided prepaid envelope. Information about the study was disclosed in the initial surveys with declaration that the return of the survey constituted as informed consent for participating in the study. This study adhered to the Declaration of Helsinki and was approved by the Indiana University Institutional Review Board.

### Respondents

Participants were categorized into two cohorts based on initial inclusion diagnoses. For respondents with strabismus collected data included sex, date of birth, race/ethnicity, birth route, whether C-section was medically recommended or elective, gestational age at birth, complications during pregnancy, and complications during labor Similarly, data collected from returned surveys of the JIA group included information about sex, date of birth, race/ethnicity, age of JIA diagnosis, medication for JIA, presence of eye disorders, birth route, whether C-section was medically recommended or elective, gestational age at birth, complications during pregnancy, and complications during birth. A comprehensive list of the data collected from each survey can be found in supplementary data. Participant responses were gathered through the surveys and recorded electronically for data consolidation.

### Survey

The survey employed a combination of free response, binary (yes/no), and multiple-choice questions. Binary questions addressed sex, birth route, and whether C-section was medically recommended or not. Multiple choice questions were utilized for pregnancy complications, labor complications, JIA medication, race/ethnicity, and eye disorders. Participants selecting ‘other’ were prompted for further details in their responses. Questions requiring further elaboration or those that were challenging to categorize were managed through free responses. Date of birth, gestational age at birth, and age of JIA diagnosis were collected through free response questions.

### Qualitative analysis

A qualitative approach was adopted to address responses necessitating further elaboration. Responses specifying medication names and complications during pregnancy or labor beyond questionnaire options underwent preliminary analysis to ensure comprehensiveness. These responses were then quantified for frequency and type during analysis.

### Statistical analysis

Descriptive analysis was applied to determine median and mode age for diagnosis and gestational age at birth. A binary methodology was used employed to calculate the occurrence of all other variables. Correlation between the variables of interest was assessed using chi-square analysis, as well as Fisher's exact test for smaller cell samples (Supplementary Table).

## Conclusion

Our investigation revealed no relationship between JIA and birth route, pregnancy complications, or labor complications, although the constrained respondent count limits data interpretation. In addition, JIA-uveitis cohorts demonstrated minimal association with variation in birth route and labor complications. However, pregnancy complications, namely gestational diabetes, showed statistical significance in uveitis development within the JIA patient population. In sum, these findings offer valuable insights into the plausible causal link between JIA patients born of gestational diabetes and uveitis. Subsequent research endeavors are needed to validate this relationship and uncover potential underlying mechanisms.

### Supplementary Information

Supplementary Table 1.

## Data Availability

The data that support the findings of this study are available from the corresponding author upon reasonable request.

## Abbreviations

JIA: Juvenile idiopathic arthritis
GD: Gestational diabetes
CS: Cesarean section
RA: Rheumatoid arthritis

## References

[1] Heiligenhaus A, Heinz C, Edelsten C, Kotaniemi K, Minden K (2013). Review for disease of the year: Epidemiology of juvenile idiopathic arthritis and its associated uveitis: The probable risk factors. Ocul. Immunol. Inflamm..

[2] Heiligenhaus A, Minden K, Föll D, Pleyer U (2015). Uveitis in juvenile idiopathic arthritis. Dtsch. Ärztebl. Int..

[3] Sahin S (2021). Frequency of juvenile idiopathic arthritis and associated uveitis in pediatric rheumatology clinics in Turkey: A retrospective study, JUPITER. Pediatr. Rheumatol. Online J..

[4] Kim G (2020). Delayed establishment of gut microbiota in infants delivered by cesarean section. Front. Microbiol..

[5] Carlens C (2009). Perinatal characteristics, early life infections and later risk of rheumatoid arthritis and juvenile idiopathic arthritis. Ann. Rheum. Dis..

[6] Kaur N, Chen C-C, Luther J, Kao JY (2011). Intestinal dysbiosis in inflammatory bowel disease. Gut Microbes.

[7] Nagao-Kitamoto H (2016). Functional characterization of inflammatory bowel disease-associated gut dysbiosis in gnotobiotic mice. Cell. Mol. Gastroenterol. Hepatol..

[8] Scher JU (2015). Decreased bacterial diversity characterizes the altered gut microbiota in patients with psoriatic arthritis, resembling dysbiosis in inflammatory bowel disease. Arthritis Rheumatol..

[9] Yoo JY, Groer M, Dutra SVO, Sarkar A, McSkimming DI (2020). Gut microbiota and immune system interactions. Microorganisms.

[10] He Z, Shao T, Li H, Xie Z, Wen C (2016). Alterations of the gut microbiome in Chinese patients with systemic lupus erythematosus. Gut Pathog..

[11] López P (2016). Th17 responses and natural IgM antibodies are related to gut microbiota composition in systemic lupus erythematosus patients. Sci. Rep..

[12] Luo XM (2018). Gut microbiota in human systemic lupus erythematosus and a mouse model of lupus. Appl. Environ. Microbiol..

[13] Lazar V (2018). Aspects of gut microbiota and immune system interactions in infectious diseases, immunopathology, and cancer. Front. Immunol..

[14] Kim CH (2021). Control of lymphocyte functions by gut microbiota-derived short-chain fatty acids. Cell. Mol. Immunol..

[15] Qian X (2020). Gut microbiota in children with juvenile idiopathic arthritis: Characteristics, biomarker identification, and usefulness in clinical prediction. BMC Genomics.

[16] Arvonen M (2016). Gut microbiota-host interactions and juvenile idiopathic arthritis. Pediatr. Rheumatol..

[17] Black M, Bhattacharya S, Philip S, Norman JE, McLernon DJ (2015). Planned cesarean delivery at term and adverse outcomes in childhood health. JAMA.

[18] Sevelsted A, Stokholm J, Bønnelykke K, Bisgaard H (2015). Cesarean section and chronic immune disorders. Pediatrics.

[19] Xiong Z (2022). Prevalence of eczema between cesarean-born and vaginal-born infants within 1 year of age: A systematic review and meta-analysis. Eur. J. Pediatr..

[20] Yerlikaya FH, EryavuzOnmaz D, Altunhan H, Ilhan M (2021). Can altered colostrum miRNA expression profile after cesarean delivery be a risk factor for autoimmune diseases?. Am. J. Reprod. Immunol..

[21] Soullane S (2021). Cesarean delivery and risk of hospitalization for autoimmune disorders before 14 years of age. Eur. J. Pediatr..

[22] Räisänen L, Viljakainen H, Sarkkola C, Kolho K-L (2021). Perinatal risk factors for pediatric onset type 1 diabetes, autoimmune thyroiditis, juvenile idiopathic arthritis, and inflammatory bowel diseases. Eur. J. Pediatr..

[23] Tamburini S, Shen N, Wu HC, Clemente JC (2016). The microbiome in early life: Implications for health outcomes. Nat. Med..

[24] Cox LM (2014). Altering the intestinal microbiota during a critical developmental window has lasting metabolic consequences. Cell.

[25] Nobel YR (2015). Metabolic and metagenomic outcomes from early-life pulsed antibiotic treatment. Nat. Commun..

[26] Yoshioka H, Iseki K, Fujita K (1983). Development and differences of intestinal flora in the neonatal period in breast-fed and bottle-fed infants. Pediatrics.

[27] Martín R (2003). Human milk is a source of lactic acid bacteria for the infant gut. J. Pediatr..

[28] Heikkilä MP, Saris PEJ (2003). Inhibition of *Staphylococcus*
*aureus* by the commensal bacteria of human milk. J. Appl. Microbiol..

[29] Pantoja-Feliciano IG (2013). Biphasic assembly of the murine intestinal microbiota during early development. ISME J..

[30] Shenoi S, Shaffer ML, Wallace CA (2016). Environmental risk factors and early-life exposures in juvenile idiopathic arthritis: A case-control study. Arthritis Care Res..

[31] Chaudhari M (2006). Impaired reproductive fitness in mothers of children with juvenile autoimmune arthropathies. Rheumatol. Oxf. Engl..

[32] Shenoi S, Bell S, Wallace CA, Mueller BA (2015). Juvenile idiopathic arthritis in relation to maternal prenatal smoking. Arthritis Care Res..

[33] Colebatch AN, Edwards CJ (2011). The influence of early life factors on the risk of developing rheumatoid arthritis. Clin. Exp. Immunol..

[34] Murray SR, Reynolds RM (2020). Short- and long-term outcomes of gestational diabetes and its treatment on fetal development. Prenat. Diagn..

[35] Babu GR (2019). Do gestational obesity and gestational diabetes have an independent effect on neonatal adiposity? Results of mediation analysis from a cohort study in South India. Clin. Epidemiol..

[36] Şanlı E, Kabaran S (2019). Maternal obesity, maternal overnutrition and fetal programming: Effects of epigenetic mechanisms on the development of metabolic disorders. Curr. Genomics.

[37] Moore BF, Harrall KK, Sauder KA, Glueck DH, Dabelea D (2020). Neonatal adiposity and childhood obesity. Pediatrics.

[38] Kim SY, Sharma AJ, Callaghan WM (2012). Gestational diabetes and childhood obesity: What is the link?. Curr. Opin. Obstet. Gynecol..

[39] Muhammad FY, Peters K, Wang D, Lee DJ (2019). Exacerbation of autoimmune uveitis by obesity occurs through the melanocortin 5 receptor. J. Leukoc. Biol..

