# Supplementary material for: Perinatal events and development of juvenile idiopathic arthritis-associated uveitis
Source: Sci Rep. 2023 Oct 16;13:17576. doi: 10.1038/s41598-023-44208-1 (PMC10579364; doi:10.1038/s41598-023-44208-1)
Supplement: Supplementary file 1 — Supplementary Table 1. [file 41598_2023_44208_MOESM1_ESM.docx]

**Supplement Table:** List of answer choices given to respondents

| **Pregnancy Complications** | **Labor Complications** | **Eye Disorders** | **Medications** |
| --- | --- | --- | --- |
| Hypertension | Breech position | Uveitis | Adalimumab |
| Gestational diabetes | Preterm rupture of membranes | Iritis | Infliximab |
| Preeclampsia | Decelerating fetal heart tones | cataracts | Methotrexate |
| Anemia | Placental abruption | Pars Planitis | Ibuprofen |
| Infection | Umbilical cord prolapse | Glaucoma | Naproxen |
| Breech position | Prolonged labor | Retinal detachment | Rituximab |
| Cigarette smoking | Post-term pregnancy | Macular edema | Prednisone |
| Alcohol use | Other | Band keratopathy | Other |
| Preterm labor |  | Other |  |
| Other |  |  |  |
